# Squamous Cell Carcinoma of the Vulva: A Survival and Epidemiologic Study with Focus on Surgery and Radiotherapy

**DOI:** 10.3390/jcm11041025

**Published:** 2022-02-16

**Authors:** Matteo Scampa, Daniel F. Kalbermatten, Carlo M. Oranges

**Affiliations:** Department of Plastic, Reconstructive and Aesthetic Surgery, Geneva University Hospitals, University of Geneva, 1205 Geneva, Switzerland; matteo.scampa@hcuge.ch (M.S.); daniel.kalbermatten@hcuge.ch (D.F.K.)

**Keywords:** vulva, squamous cell carcinoma, survival, epidemiology, SEER

## Abstract

Vulvar squamous cell carcinoma (SCC) is the most frequent vulvar neoplasia. While the primary role of surgery is widely accepted, large population studies are needed to compare survival between diverse treatment modalities and to identify independent prognostic factors to help council patients and guide oncological treatment. The U.S. National Cancer Index, Surveillance, Epidemiology and End Results (SEER) program data between 2000 and 2018 was screened for all squamous cell carcinoma affecting the vulva. Raw data was processed with IBM SPSS. Demographic, clinical-pathological and treatment data were studied. Overall survival (OS) was calculated using the Kaplan–Meier method and subgroups were compared using the log rank test. A multivariate cox regression was conducted to identify independent prognostic factors. A total of 11,360 patients were identified with a median age of 65. Median overall survival was 101 months. Surgery as a primary treatment is the therapeutic sequence associated with the best overall survival. Multivariate cox-regression did not meet proportional hazard assumption. Age, pathological grade, stage at diagnosis, treatment sequence and the use of chemotherapy were identified as independent prognostic factor. Surgery alone is the treatment sequence offering the best overall survival. Surgery should be offered to all eligible patients.

## 1. Introduction

Squamous cell carcinoma (SCC) can affect a large number of organs covered by squamous epithelial lining such as the vulva skin and the vagina [1]. Vulvar carcinoma is one of the less common gynaecological neoplasia with incidence as low as 2.6 per 100,000 person per year [2,3]. SCC represents the majority of vulvar cancer. Vulvar SCC can have different clinical presentations such as ulcerated plates or wart-like lesions with different colorations reported [4]. The carcinogenesis can occur with two different pathways. High-risk human papillomavirus (HPV) infection, despite its known role in cervical cancer, can also trigger vulvar SCC. Individuals tend to be young patients whereas HPV-independent SCC usually occurs in older individuals with chronic inflammatory background such as lichen sclerosis [5]. Precursor lesions have been identified as squamous intraepithelial lesion (SIL), typically associated with papillomavirus infection, and differentiated vulvar intraepithelial neoplasia (dVIN), usually associated with chronic inflammation. The two precursors present different characteristics and behaviour [6,7]. 

Due to invasive behaviour, SCC requires a multidisciplinary approach to limit disease burden, recurrence and mortality. In current guidelines, surgery maintains a main role in local and regional control. Use of adjunctive treatments such as radiotherapy is frequent [8]. Radical vulvectomy and radical local excision can lead to extensive soft tissues defects, local function impairment (urinary or faecal incontinence) and sexual disfunctions [9]. Plastic surgeons maintain an important role by providing local reconstruction allowing restoration of function and cosmetic improvement. Early detection allows to treat the neoplasia at a localized stage with less invasive surgery. Large population studies that focus on epidemiology and survival are needed to improve surgical care and identify prognostic factors. The Surveillance, Epidemiology and End Results (SEER) program of the National Cancer Institute (NCI) is an incidence and survival database that covers up to 35% of the USA and allows large population study for low incidence tumours [10].

Studies assessing the SEER database for data on vulvar carcinoma were conducted, but to our knowledge none focused specifically on SCC and its association between demographics, treatment and overall survival [11,12,13,14].

The aim of our study is to offer recent epidemiologic and survival data over the vulvar SCC by focusing on surgery and radiation therapy to improve surgical care and help guide patient counselling.

## 2. Materials and Methods

Patient selection: The NCI SEER database including data between 2000 and 2018 through 18 registries was screened for all squamous cell carcinoma using ICD-O3 histopathological codes 8070 to 8085. Primary topographic site was selected using ICD codes for Labium Majus (C51.0), Labium Minus (C51.1), Clitoris (C51.2), Overlapping lesion of vulva (C51.8) and Vulva, NOS (Not Otherwise Specified) (C51.9). 

Analysis: Raw data were extracted using the case listing function of the survival session of the Surveillance Research Program, National Cancer Institute SEER*Stat software (seer.cancer.gov/seerstat, accessed on 30 December 2021) version 8.3.9 and processed through IBM SPSS v.27 (IBM, Armonk, NY, USA). Epidemiologic and clinical-pathological characteristics of patients were described. Overall survival (OS) was calculated using the Kaplan–Meier method and was defined as the time from diagnosis to death regardless of the cause. Log-rank test was used to compare OS between subgroups. Multi-variate Cox regression was used to identify independent prognostic factors. To validate results, a proportional hazards (PH) assumption test was conducted using Schoenfeld’s residuals. A *p*-value of <0.05 was considered statistically significant. Unknown or missing data were not included in statistical analysis.

Variable selection: SEER variables were used when available (age, race, marital status, stage at diagnosis, type of surgery, radiotherapy and chemotherapy), but some were created using the merging function of the SEER*stat software that allows to combine different variables (pathological grade, treatment sequence). For comparing OS between age groups, 3 categories were created: <65 years old (y.o.); 65–79 y.o.; ≥80 y.o. Age subdivision in three categories was performed by randomly assuming patients younger than 65 were expected to be fit and active with a better prognosis, whereas patient of/over 80 years old had more comorbidities and a lower OS was expected. Stage at diagnosis was based on the SEER summary stage where localized disease is defined as an invasive carcinoma with or without stromal invasion confined to musculature, submucosa and vulva including the skin. Regional disease is characterized by direct extension to adjacent perineal structures such as the anus, peri-anal skin, bladder, distal third urethra, rectum and vagina or/and regional lymph nodes involvement. Distant disease includes further extension such as bladder or rectal mucosa, rest of urethra (proximal 2/3), pelvic bone, perineal body and/or distant lymph nodes. Further details are available on the SEER program website [15]. Since 2018, the pathological grade of tumour reported in the SEER database uses a 3-category classification, meaning Grade IV (anaplastic tumours) were combined with Grade III (poorly differentiated). To ease results interpretation, we converted all data from before 2018 in this 3-stage system. When assessing histologic sub-types, we considered for analysis only sub-types that represented more than 1% of the study population because most of the 13 subtypes included less than 100 patients. Treatment sequence variable was created by merging the surgery and radiation variables. It included 4 subgroups, where surgery included all kind of surgery and even unknown surgery if documented as surgery unknown. Radiotherapy included all kind of radiation therapy without distinction between neo-adjuvant and adjuvant.

## 3. Results

**Figure 1:**
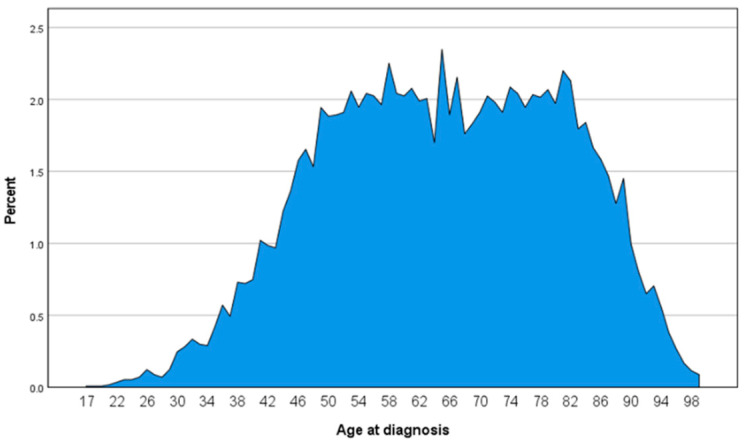
Diagnosis age distribution of vulvar squamous cell carcinoma.

**Figure 2:**
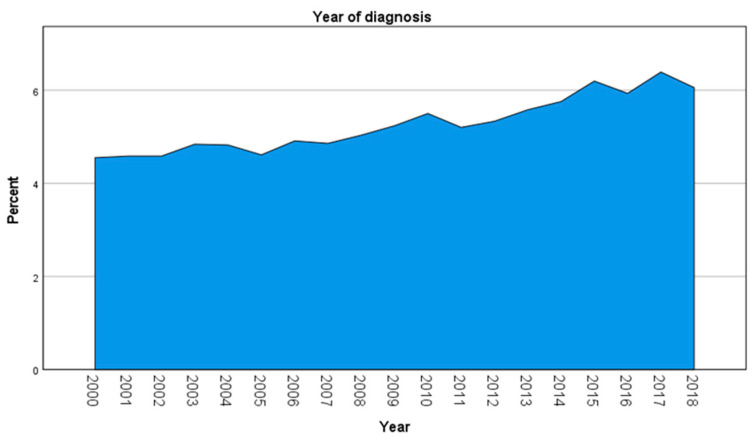
Distribution of vulvar squamous cell carcinoma cases through the years.

A cohort of 11,360 patients was identified (Table 1). Median age at diagnosis was 65 years with a range from 17 to 99 years (Figure 1). Between 2000 and 2018, a small increase in cases diagnosed by year has been observed through the years (Figure 2). When assessing demographic and clinical–pathological characteristics, we noted that most of the patient were white married women with a localized disease. Vulva, not otherwise specified (NOS) was reported as the most frequent primary site.

Thirteen different histologic subtypes were described, but 68.1% were categorized under squamous cell carcinoma, NOS. The mean initial tumour size was 33.5 mm (σ = 37.6; median 27). Most patients had a moderately differentiated pathological grade (G2). Surgery was performed in 80.9%, with simple/partial surgical removal of primary site being the most reported category (33.7%). A minority of patients received radiation therapy (30.6%) and the use of chemotherapy was rare (18.3%). A total of 3247 patients (93.5%) were treated with beam radiation, 121 (3.5%) with a combination of beam radiation with implants or isotopes, 27 patients (<1%) with radioactive implants or radioisotopes. The majority of patients received radiotherapy after surgery (2035 patients), 237 patients received radiotherapy prior to surgery and 27 received radiation therapy before and after surgery. For patients that did not benefit from surgery the main reason reported was “not recommended”.

Median OS was 101 months (m) (95% CI {96.4–105.6}). Five-year OS was 60.6%; 10 years OS was 45.7%; and 15 years OS was 33.1%.

**Figure 3:**
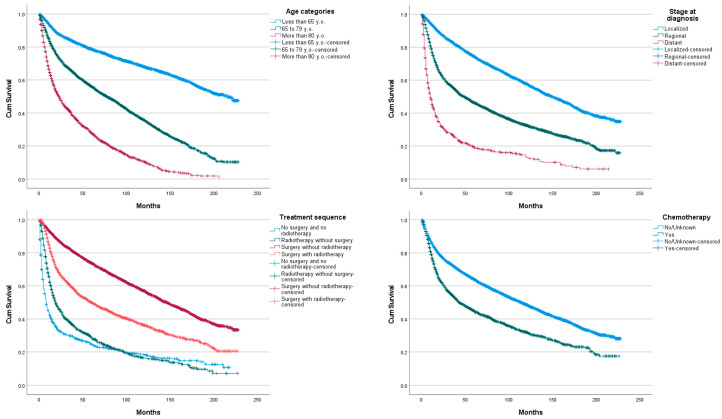
Vulvar squamous cell carcinoma overall survival according to age, stage at diagnosis, treatment sequence and use of chemotherapy.

**Figure 4:**
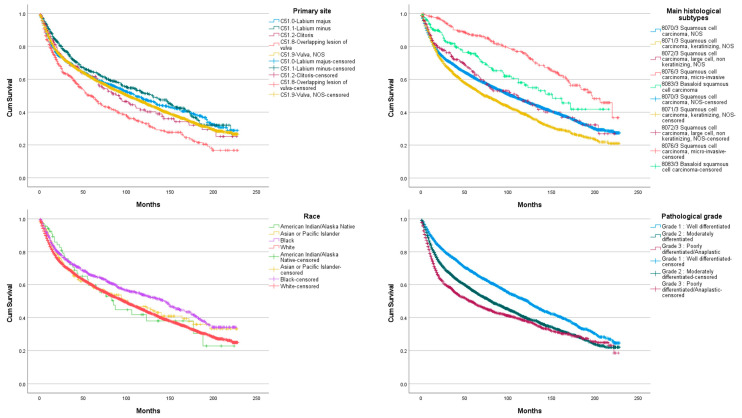
Vulvar squamous cell carcinoma overall survival according to primary site, histological subtype, race and pathological grade.

When assessing OS between three categories of age we found statistical difference (*p* < 0.05) between each group (Figure 3). Between all primary site, overlapping lesion of the vulva was found to have the lowest OS compared to other sites (*p* < 0.05). We also found statistical difference between Vulva, NOS and labium minus. Other sites, however, did not differ significantly in terms of OS (Figure 4). According to histological subtype, squamous cell carcinoma, keratinizing, NOS (ICD-O3 8071/3) was found to have the lowest OS (*p* < 0.05). Squamous cell carcinoma, micro-invasive (ICD-O3 8076/3), followed by basaloid squamous cell carcinoma (ICD-O3 8083/3) were found to have significantly better OS than other histological subtypes (*p* < 0.05). However, no statistical difference was found between squamous cell carcinoma, NOS (ICD-O3 8070/3) and squamous cell carcinoma, large cell, non-keratinizing, NOS (ICD-O3 8072/3).

When assessing OS between races, we found a statistical difference between white and black populations with the later having a better OS. OS significantly lowered with pathological grade increasing (*p* < 0.05). Regional and distant invasion at diagnosis is associated with lower OS (*p* < 0.05). Surgery without radiotherapy was the treatment sequence associated with the better OS, followed by surgery with radiotherapy, radiotherapy without surgery and no surgery and no radiotherapy. OS differences between each sequence was statistically significant (*p* < 0.05). The use of chemotherapy was associated with low OS (*p* < 0.05).

Cox regression included 8606 cases. A total of 2754 cases were dropped due to missing data, corresponding to 24.2% of the total population. With the multivariate cox-regression we identified age, some of the main histological subtypes, pathological grade, some of the races, stage at diagnosis, treatment sequence and the use of chemotherapy as independent prognostic factors of improved survival (Table 2). Surgery without radiotherapy presented a significant hazard ratio of 0.227 (95% CI {0.200–0.258}) compared to no surgery and no radiotherapy. However, age at diagnosis, main histological subtype, pathological grade, treatment sequence and chemotherapy did not meet the proportional hazard assumption necessary to validate the Cox regression model. Caution should be employed when interpreting prognostic factors.

## 4. Discussion

Our study identified more than 11,000 vulvar SCC cases and is to our knowledge the biggest survival study population [11,16,17,18,19,20]. We found that vulvar SCC affects a majority of white women with disease mostly diagnosed at a localized stage. Those results affirm current knowledge [16,17,18,19,20]. Pathological grade 2 (moderately differentiated) is also reported as the most frequent by several studies [16,20]. Age at diagnosis seems to be younger in our population compared to other studies [16,17,19,20]. Elderly patients have the lowest survival of the three categories.

In our study more than 60% of our population benefit from surgery as the sole treatment modality. Results confirms current literature where surgery alone is the most frequently reported treatment sequence followed by its association with radiotherapy and finally radiotherapy alone [16,19,20]. Rottman et al. reported partial vulvectomy, followed by wide excision as the most frequent procedures [20]. Comparison with our results is limited because of different designations in the SEER database, but the trend is similar with simple/partial surgical removal of primary site, followed by “en bloc” resection being the most frequent procedures. Results stay in line with current recommendations. The National Comprehensive Cancer Network recommends wide local excision for localized disease with less than 1mm invasion and radical local excision or modified radical vulvectomy with lymph node assessment (sentinel lymph node or nodal dissection) for localized disease with more than 1 mm invasion. For advanced diseases (locally advanced and regional), lymph node control maintains an important role and may require adjuvant radiotherapy and/or chemotherapy [14]. Distant disease requires multimodal palliative care [8]. 

Interestingly in our population 5-year OS was better than the one reported by Hellman in Sweden and Rottmann in Germany (59% and 55%, respectively), this could be correlated to the older median age reported by both studies because age has been identified as an independent prognostic factor in our study [16,20]. Surgery without radiotherapy has been identified as the treatment offering the best OS in the study population. This result seems to be especially true for localized disease (most of the study patients) where it has been recommended as standalone treatment. However, its role as a standalone treatment in more advanced disease is questionable with regional disease OS being lower. Surgery associated with radiotherapy is currently recommended for diseases with nodal involvement. Gill suggested that the concomitant use of adjuvant chemotherapy to adjuvant radiotherapy in patient with nodal involvement improved overall survival [21].

Radiotherapy alone, however, did not offer drastic improvement in survival compared to no treatment, except a small improvement in the first year compared to the later. Stecklein suggested that radiotherapy as a sole treatment might have a role in regional control for locally advanced tumours deemed unresectable [22]. 

Interestingly in our study we found that the overall survival of patient that benefited from chemotherapy was statistically lower from those who did not benefit from it. This observation can be explained by an indication bias: chemotherapy is used mainly in patients with advanced disease, with a lower survival. Rao et al, suggested that chemotherapy associated with radiotherapy was superior to radiotherapy alone in patients that did not benefit from surgery [23]. Reade et al, suggested in their review that while some studies suggest a positive impact of chemoradiation, the use of chemotherapy as a sole treatment remains questionable in the absence of new studies due to limited survival and high treatment toxicity [24]. Despite a non-valid Cox regression model due to PH assumption not being met, our results showed similar results to the VULCAN study who also identified chemotherapy as an independent prognostic factor of improved survival [25].

The role of adjuvant and neo-adjuvant therapies is believed to be mainly relevant in regional or distant disease. However, as Mazzotta et al. suggested in their review, the level of evidence remains low and further research must be conducted to assess their real impact on survival and recurrence and to define the optimal tailored treatment strategy [26].

Survival analysis according to treatment sequences should be interpreted cautiously as indication bias can be frequent, as seen for the use of chemotherapy in this article. It is one of the main limitations of the SEER database, which does not report the scope of treatment. The aim of lymph node procedures was not specified (diagnostic vs. curative) and their analysis was not conducted to avoid potential bias. Furthermore, some variables such as the positive lymph node count and their location were incomplete and not included in our analysis.

Another limitation of our study was the incapacity to integrate HPV status in our survival data because it was not reported in SEER. HPV positive SCC seem to have a better overall survival compared to HPV-independent SCC and non-surgical treatment might have a different impact [6,27,28,29]. Further studies assessing survival according to different treatment sequences in HPV positive and negative patients are required.

## 5. Conclusions

Vulvar SCC is a rare disease affecting women around their sixties and mainly diagnosed at a localized stage. Outcome is mainly determined by the age of the patient, pathological grade, stage at diagnosis and the choice of treatment. We found that surgery maintains a primordial role in primary disease control and offer the best overall survival compared to other treatment sequences. Surgery should be offered to all eligible patients. 

Further studies should be conducted to assess survival and recurrence outcomes between different treatment modalities and different stage of disease.

## Figures and Tables

**Table 1:** Demographic and clinical-pathological features of vulvar squamous cell carcinoma.

	N (%)
Age	
Median	65 (range 17–99)
<65 years old	5440 (57.9)
65 to 79 years old	3409 (30)
≥80 years old	2511 (22.1)
Race	
White	9895 (87.1)
Black	1045 (9.2)
American Indian/Alaska Native	69 (0.6)
Asian or Pacific Islander	255 (2.2)
Unknown	96 (0.8)
Marital status	
Married	4179 (36.8)
Not Married	3587 (31.6)
Widowed	2745 (24.2)
Unknown	849 (7.5)
Primary site	
C51.0 Labium majus	965 (8.5)
C51.1 Labium minus	536 (4.7)
C51.2 Clitoris	211 (1.9)
C51.8 Overlapping lesion of vulva	521 (4.6)
C51.9 Vulva, NOS *	9127 (80.3)
Histologic subtypes	
8070/3 Squamous cell carcinoma, NOS *	7740 (68.1)
8071/3 Squamous cell carcinoma, keratinizing, NOS *	2889 (25.4)
8072/3 Squamous cell carcinoma, large cell, non-keratinizing, NOS *	248 (2.2)
8076/3 Squamous cell carcinoma, micro-invasive	266 (2.3)
8083/3 Basaloid squamous cell carcinoma	173 (1.5)
Pathologic grade	
G1: Well differentiated	2900 (25.5)
G2: Moderately differentiated	4317 (38)
G3: Poorly differentiated/Anaplastic	1761 (15.5)
Unknown	2382 (21)
Stage at diagnosis	
Localized	6425 (56.6)
Regional	3897 (34.3)
Distant	664 (5.8)
Unknown	374 (3.3)
**No surgery**	2122 (18.7)
**Surgery**	9204 (80.9)
Local destruction	13 (0.1)
Local excision	1591 (14)
Simple/partial surgical removal of primary site	3831 (33.7)
Total surgical removal of primary site	1405 (12.4)
Debulking surgery	25 (0.2)
En bloc resection	2277 (20)
Surgery, NOS *	62 (0.5)
**Unknown**	34 (0.3)
Radiotherapy	
Yes	3473 (30.6)
No/unknown	7886 (69.4)
Treatment	
No surgery and no radiotherapy	745 (6.6)
Radiotherapy without surgery	1353 (11.9)
Surgery without radiotherapy	7028 (61.9)
Surgery with radiotherapy	2115 (18.6)
Chemotherapy	
Yes	2079 (18.3)
No/unknown	9281 (81.7)

* NOS: Not Otherwise Specified.

**Table 2:** Multivariate Cox-regression of vulvar squamous cell carcinoma.

	B	Sig.	Exp(B)	95% CI for Exp(B) Low/Up
Age: 3 subgroups				
Less than 65 y.o.		0.000		
65 to 79 y.o.	0.936	0.000	2.550	2.360–2.756
More than 80 y.o.	1.647	0.000	5.193	4.790–5.631
Primary site				
Labium majus		0.472		
Labium minus	0.066	0.446	1.068	0.902–1.264
Clitoris	0.139	0.235	1.149	0.914–1.444
Overlapping lesion of vulva	0.132	0.102	1.141	0.974–1.337
Vulva, NOS	0.084	0.123	1.087	0.978–1.209
Main histological subtype				
8070/3 Squamous cell carcinoma, NOS		0.000		
8071/3 Squamous cell carcinoma, keratinizing, NOS	0.123	0.000	1.131	1.057–1.211
8072/3 Squamous cell carcinoma, large cell, non-keratinizing, NOS	−0.325	0.003	0.722	0.583–0.895
8076/3 Squamous cell carcinoma, micro-invasive	−0.246	0.189	0.782	0.541–1.129
8083/3 Basaloid squamous cell carcinoma	−0.273	0.075	0.761	0.564–1.028
Pathological grade				
Grade 1: Well differentiated		0.000		
Grade 2: Moderately differentiated	0.153	0.000	1.165	1.083–1.253
Grade 3: Poorly differentiated/Anaplastic	0.285	0.000	1.330	1.215–1.456
Stage at diagnosis				
Localized		0.000		
Regional	0.524	0.000	1.689	1.568–1.820
Distant	1.196	0.000	3.308	2.913–3.755
Race				
American Indian/Alaska Native		0.000		
Asian or Pacific Islander	−0.671	0.001	0.511	0.339–0.772
Black	−0.423	0.028	0.655	0.450–0.955
White	−0.274	0.136	0.760	0.530–1.090
Treatment sequence				
No surgery and no radiotherapy		0.000		
Radiotherapy without surgery	−0.577	0.000	0.562	0.484–0.652
Surgery without radiotherapy	−1.482	0.000	0.227	0.200–0.258
Surgery with radiotherapy	−1.162	0.000	0.313	0.273–0.359
Chemotherapy				
No/Unknown				
Yes	−0.190	0.000	0.827	0.750–0.910

## Data Availability

Raw data available on the SEER program https://seer.cancer.gov/ (accessed on 30 December 2021).
